# Efficiency Models for GaN-Based Light-Emitting Diodes: Status and Challenges

**DOI:** 10.3390/ma13225174

**Published:** 2020-11-17

**Authors:** Joachim Piprek

**Affiliations:** NUSOD Institute LLC, Newark, DE 19714-7204, USA; piprek@nusod.org

**Keywords:** InGaN/GaN, light-emitting diode, efficiency droop, drift-diffusion, leakage, Auger recombination, light extraction

## Abstract

Light-emitting diodes (LEDs) based on Gallium Nitride (GaN) have been revolutionizing various applications in lighting, displays, biotechnology, and other fields. However, their energy efficiency is still below expectations in many cases. An unprecedented diversity of theoretical models has been developed for efficiency analysis and GaN-LED design optimization, including carrier transport models, quantum well recombination models, and light extraction models. This invited review paper provides an overview of the modeling landscape and pays special attention to the influence of III-nitride material properties. It thereby identifies some key challenges and directions for future improvements.

## 1. Introduction

The 2014 Nobel Prize in Physics was awarded jointly to Isamu Akasaki, Hiroshi Amano and Shuji Nakamura *“for the invention of efficient blue light-emitting diodes which has enabled bright and energy-saving white light sources”* [1]. Their demonstration of GaN-based light-emitting diodes (LEDs) triggered intense worldwide research and development efforts, not only for general lighting applications, but also in many other areas, such as displays, biotechnology, sensing, and medical instrumentation.

**Figure 1:**
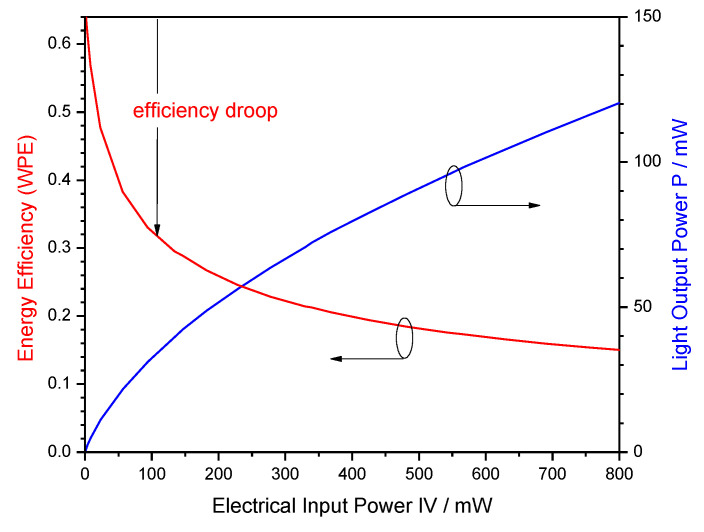
Illustration of the energy efficiency (wall-plug efficiency WPE) as ratio of output power to input power.

**Figure 2:**
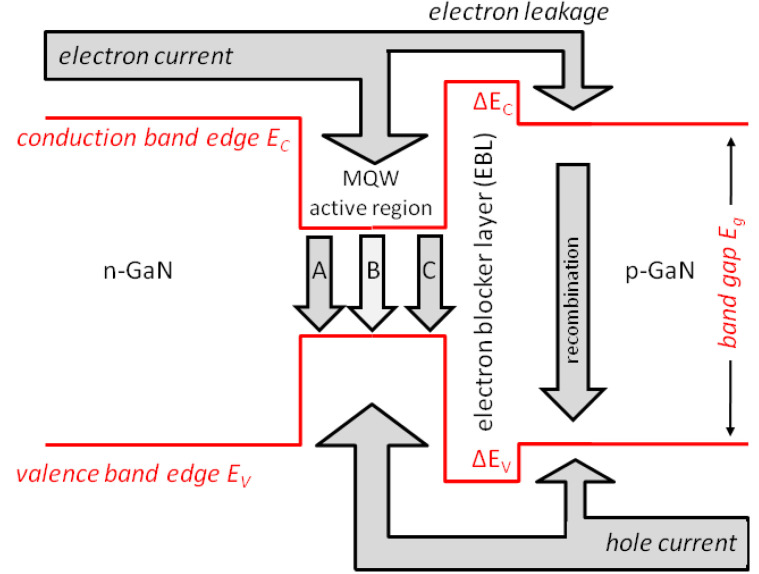
Illustration of the vertical LED energy band diagram including current flow and electron–hole recombination processes (A—defect-related recombination, B—photon emission, C—Auger recombination) [4]. Multiple quantum well (MQW) active regions typically consist of InGaN wells and GaN barriers. The electron blocker layer (EBL) is typically made of AlGaN.

The promise of superior energy efficiency is the main driving force of many research activities on GaN-LEDs [2,3]. However, high efficiency is only observed at low injection current density and low power (Figure 1). With rising current, injected electron–hole pairs disappear increasingly in parasitic processes without generating light, thereby causing severe efficiency droop [4]. Still debated is the specific non-radiative mechanism that dominates this efficiency droop, which may be different in different devices. The two leading explanations are Auger recombination inside the light-generating InGaN quantum wells (QWs) [5] and electron leakage into p-doped layers [6], respectively, in possible combination with other effects (see Figure 2 for illustration). However, very few direct measurements of either mechanism are published, none of which establishes a dominating magnitude. Most publications on efficiency droop mechanisms base their quantitative claims on modeling and simulation [4,7,8]. Nevertheless, the total energy efficiency is usually of greater importance [9] and it is the focus of this paper.

The energy efficiency of the LED semiconductor chip is equivalent to the so-called wall-plug efficiency (WPE) which gives the ratio of light power P emitted from the chip to electrical power IV injected into the chip (I—injected electron–hole current, V—bias) as illustrated in Figure 1. Different energy loss mechanisms reduce the WPE, which are distinguished by splitting the WPE into separate components:

WPE = ELE × EQE = ELE × IQE × LEE = ELE × IE × RE × LEE(1)

First, the injected electrons lose some energy on their way to the QWs, which is accounted for by the electrical efficiency ELE = hν/qV (hν—photon energy, q—electron charge). The remaining external quantum efficiency EQE = WPE/ELE is the ratio of emitted photon number to injected number of electron–hole pairs. The conversion of electron–hole pairs into emitted photons is accompanied by carrier losses and by photon losses: EQE = IQE × LEE. The light extraction efficiency LEE accounts for photon losses due to internal light reflection and absorption. The internal quantum efficiency IQE is the fraction of the total current that contributes to the desired photon generation inside the QWs. It can be further separated into the injection efficiency IE (current fraction that enters the QWs) and the radiative efficiency RE (fraction of QW carriers that recombines radiatively).

While WPE can be measured, the analysis of energy loss mechanisms depends mainly on modeling and simulation. For more than a decade, various models have been published for each GaN-LED efficiency component. Emphasizing the influence of material properties, we separate these models in the following into carrier transport models, QW recombination models, light extraction models, and self-heating models.

## 2. Carrier Transport Models

Drift-diffusion models based on the semiconductor transport equations are commonly employed for simulating the carrier movement of electrons and holes in GaN-LEDs [10,11]. Carrier mobilities are crucial material properties in such models, besides recombination coefficients which are covered in the next section. Together with the free carrier density, the mobility determines the conductivity of each semiconductor layer. The low hole conductivity of Mg-doped III-nitride semiconductors typically dominates the LED bias. Incomplete Mg acceptor ionization is an important but often neglected aspect of drift-diffusion models [12]. Due to the large Mg acceptor ionization energy, high Mg doping densities are required which in turn limit the free hole mobility by impurity scattering. Advanced models for carrier transport parameters have been developed [13]. Nevertheless, the material quality of fabricated devices is often best represented by experimental data, especially in the case of alloy layers. The semiconductor–metal contact may also have a strong impact on the measured bias which is hard to predict [14,15].

Several groups employ Monte-Carlo transport models to track the path of individual carriers using tailored scattering models, partially in combination with drift-diffusion models [16,17]. In particular, the movement of high-energy (hot) electrons has been investigated this way.

Quantum mechanical transport models based on the Non-Equilibrium Green’s Function (NEGF) method have been published more recently [18,19]. Such models are especially valuable in the investigation of tunneling and carrier leakage processes. However, the inclusion of electron–hole recombination is difficult and still under development [20]. Simplified tunneling models have been implemented in drift-diffusion simulations to investigate multi-quantum barriers [21], trap-assisted interband tunneling [22], or LED structures with tunnel-junction cascaded active regions [23,24].

**Figure 3:**
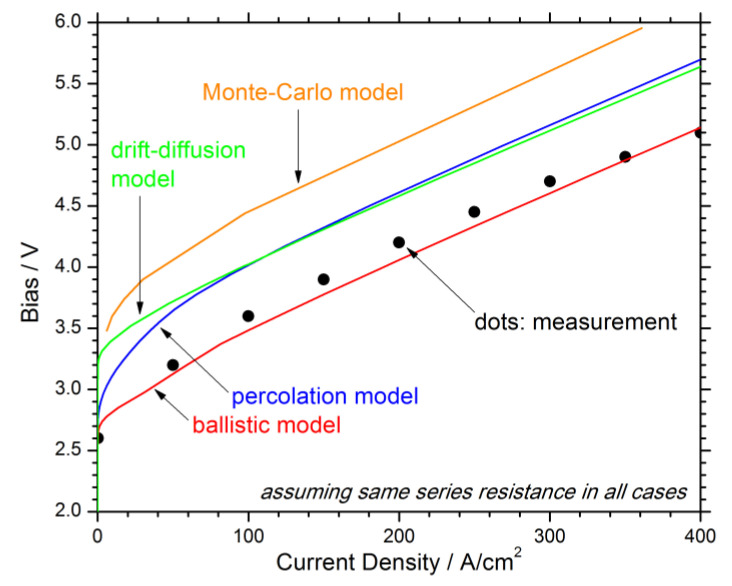
Comparison of bias-current characteristics calculated for the same LED structure with different transport models (see text).

In an organized effort to demystify the efficiency droop, different transport models were applied to the same experimental LED structure [25], including the common drift-diffusion concept [21], the Monte-Carlo method [26], the NEGF method [18], a ballistic transport model [27], and percolation transport considering random alloy fluctuations [28]. While the normalized efficiency droop was fairly well reproduced in all cases, its physical interpretation is quite different. Some authors conclude dominant Auger recombination, others observe significant electron leakage. Figure 3 shows different current-voltage (IV) curves calculated for this blue LED with a measured turn-on bias of 2.6 V [25]. The highest calculated turn-on bias of 3.5 V is obtained by the Monte-Carlo model. The common drift-diffusion model gives a turn-on bias of 3.2 V. The percolation model results in a soft turn-on starting at 2.8 V because carriers search along each interface for the lowest energy barrier. Only the ballistic transport model accounting for high-energy electrons allows for a close fit of the measured IV characteristic. Trap-assisted tunneling was not included in this comparison, which is also known to lower the turn-on bias [22].

**Figure 4:**
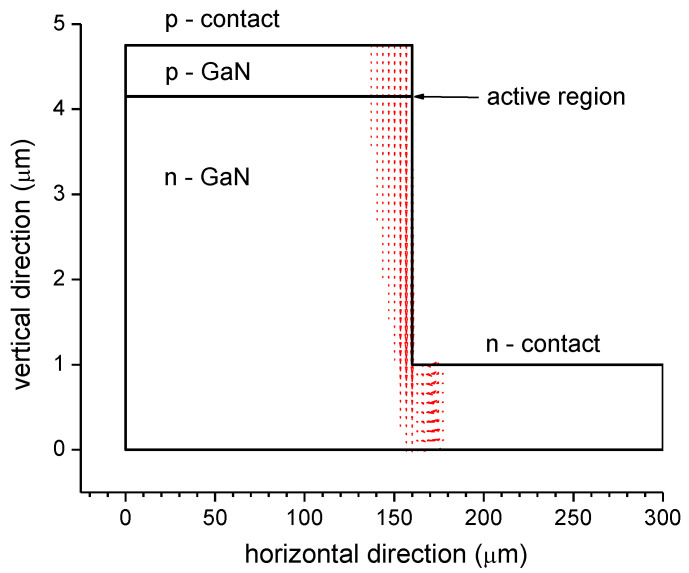
Current distribution (red arrows) calculated in a two-dimensional LED simulation (the arrow size scales with the local current density so that arrows are only visible in high-current regions) [31].

Such IV discrepancies may be caused by the employment of different material parameters. A key parameter is the Mg acceptor density inside the Mg-doped AlGaN electron blocker layer (cf. Figure 2), as only an unknown fraction of Mg atoms form AlGaN acceptors. This crucial but largely ignored p-AlGaN doping effect creates much uncertainty in GaN-LED simulations [29]. Current crowding [11] and non-uniform carrier injection into the quantum wells [10] is also often neglected or insufficiently analyzed, as most carrier transport simulations are one- or two-dimensional (2D). Figure 4 illustrates a severe case of current crowding (red arrows) observed in a 2D simulation along the vertical edge between p-contact and n-contact which is mainly caused by the low p-GaN hole conductivity and which results in a highly non-uniform current injection into the active region. However, the 3D current distribution in real devices is different and it depends on the actual conductivity of each layer [15,30].

The simulated wall-plug efficiency WPE is affected by transport models in two different ways. Firstly, the electrical efficiency ELE depends on the total device bias calculated. Figure 3 demonstrates the bias discrepancy between different modeling approaches. Luckily, the measured device bias can be used in most practical cases to determine the electrical efficiency from the observed photon emission wavelength. In fact, ELE > 1 has been measured on highly optimized GaN-LEDs [32], which is attributed to the absorption of lattice thermal energy by injected carriers before they generate photons [33], encouraging the concept of electroluminescent cooling [34].

**Figure 5:**
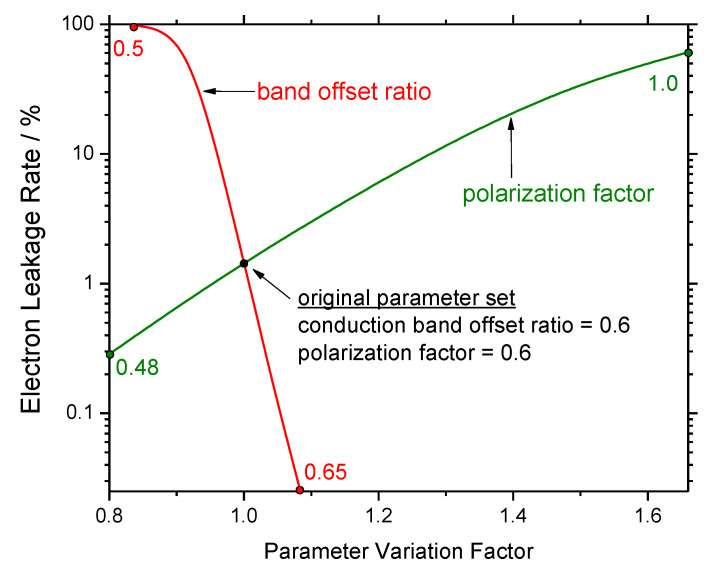
The calculated electron leakage is extremely sensitive to variations of the conduction band offset ratio ΔE_c_/ΔE_g_ (cf. Figure 2) and the scaling factor applied to the theoretically predicted material polarization field of the electron blocker layer [38].

Secondly and most importantly, transport models are essential in determining the injection efficiency IE in Equation (1), i.e., the fraction of carriers that recombines inside the quantum wells, which cannot be measured that easily. Electron leakage into p-doped layers is frequently blamed for the efficiency droop. Such leakage is most often attributed to incomplete carrier capture by the quantum wells [35] or to thermionic emission from the quantum wells [6], and less often to hot electrons [27] or to tunneling [18]. Electrons leaking into the p-doped side of the LED recombine there with holes before those holes can reach the active layers (cf. Figure 2). In other words, electron leakage and reduced hole injection are two sides of the same process. In fact, some authors consider the low hole conductivity of p-doped layers the key reason for the electron leakage [36]. The magnitude of the electron leakage was also found to be highly sensitive to other properties of the AlGaN electron blocker layer (EBL) [37,38]. Figure 5 plots the relative leakage as function of the built-in polarization and the EBL conduction band offset ratio ΔE_c_/ΔE_g_ (cf. Figure 2). Trouble is, both material parameters are not exactly known. Consequently, almost all of the many published simulation studies on EBL design and optimization are quite speculative as long as the leakage current is not validated experimentally. Only very few publications provide such experimental evidence, and none has been able to demonstrate that the magnitude of leakage fully explains the magnitude of the efficiency droop [39,40].

## 3. Quantum Well Carrier Recombination Models

Electrons and holes injected into the quantum wells of the LED can be consumed by different recombination mechanisms [41]:

A. crystal defect related recombination
B. radiative recombination
C. Auger recombination

**Figure 6:**
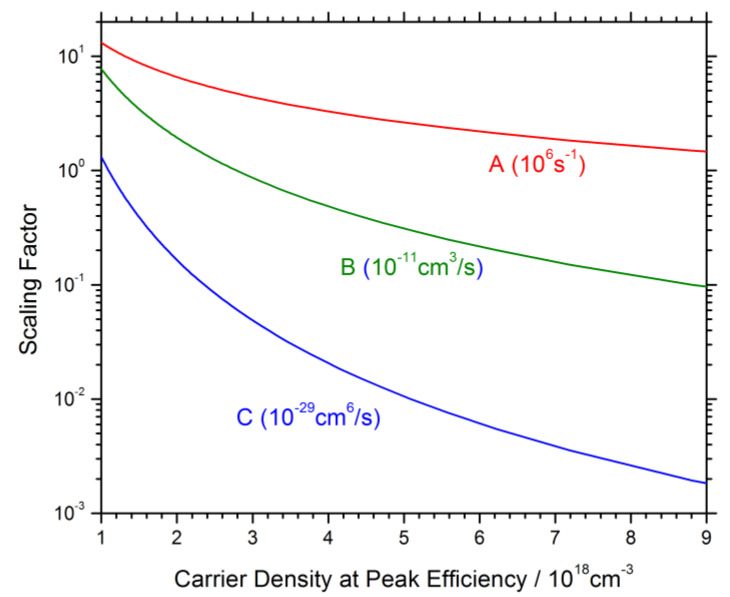
Illustration of recombination parameter sensitivity to the quantum well carrier density at peak efficiency. Each vertical combination of the recombination coefficients A, B, and C results in identical efficiency vs. current characteristics (the units are different as shown in parentheses) [42].

Accordingly, the simple and popular ABC model adds up these different contributions to the total recombination rate R(n) = A·n + B·n^2^ + C·n^3^ (n—QW carrier density; A, B, C—material parameters, cf. Figure 6) and the net current density injected into the QWs j(n) = q·d·R(n)·(d—total active layer thickness). The radiative efficiency is then given by

RE(n) = B·n^2^/(A·n + B·n^2^ + C·n^3^) (2)

However, the actual QW carrier density n is typically unknown so that different ABC parameter sets lead to identical efficiency characteristics RE(j) as illustrated in Figure 6 [42]. In fact, the QW carrier density is known to be non-uniform across a multi-quantum well active region and may even vary inside each QW due to current crowding and/or QW non-uniformities. Various groups proposed modified ABC models, e.g., to account for a reduced active volume [43], inhomogeneous carrier distribution [44], electron leakage [36,45], photon quenching [46], multi-level defects [47], trap-assisted Auger recombination [48], built-in fields [49], or temperature effects [45,50,51]. In any case, ABC models serve as an important bridge between experiment and theory [52]. More detailed models for each of the recombination mechanisms are discussed below.

(A) Defect-Related Recombination

The influence of defect-related Shockley-Read-Hall (SRH) recombination on the LED efficiency is undisputed, but it dominates only at low current or in LEDs of poor growth quality with high defect density. Instead of the parameter A in Equation (2), advanced models typically employ SRH lifetimes for electrons and holes as material parameters which can be linked to the density of defects or dislocations [53]. Crystal defects seem unable to cause any efficiency droop since the linear term (An) does not increase faster with the carrier density than the light emission (Bn^2^). For such droop to happen, the A coefficient itself must rise with the carrier density in a super-linear way. In other words, the defect-related carrier lifetime needs to decrease rapidly with higher carrier density. Some authors envisioned that QW recombination centers are located on an energy “mountain” so that they can only be reached after the QW “flatland” is filled up with carriers [54]. Other authors put this idea into a numerical model and called it Density Activated Defect Recombination (DADR) [55]. The DADR model shows good agreement with efficiency measurements at low currents, all the way down to very low temperatures. However, it fails to reproduce the efficiency droop measured at higher currents. The same is true for a band tail localization model [56] and a droop model based on the influence of QW barrier states [57]. A field-assisted SRH recombination model was proposed to explain the observed temperature sensitivity [58]. Nevertheless, all these models need to include Auger recombination or electron leakage to fully reproduce droop measurements.

(B) Spontaneous Recombination (Photon Emission)

**Figure 7:**
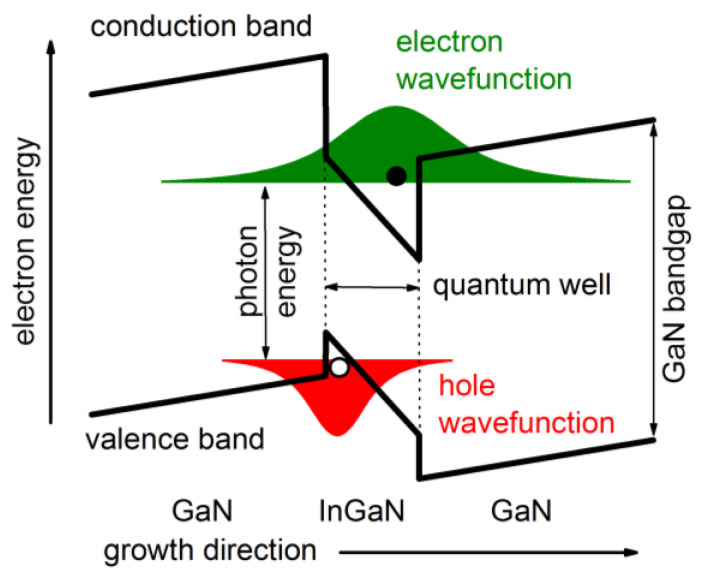
Illustration of polarization effects on an InGaN/GaN quantum well in the common Ga-polar growth direction (N-polar growth reverses the polarization field [69]).

**Figure 8:**
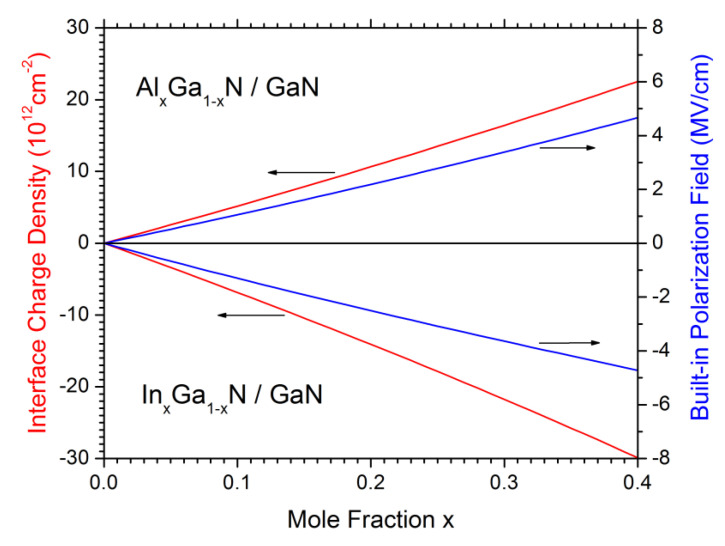
Net interface charge density and electrostatic field resulting from built-in III-nitride polarization [31]. InGaN grown on GaN results in a negative interface charge (cf. Figure 7) while growing AlGaN on GaN gives a positive interface charge.

Photon emission from InGaN/GaN quantum wells is handicapped by the built-in polarization field that separates electrons and holes inside the QW (Figure 7) thereby reducing energy and probability of spontaneous recombination (quantum confined Stark effect) [59]. Advanced GaN-LED models therefore employ a self-consistent combination of Schrödinger equation and Poisson equation in order to compute the light emission spectrum from the QW energy band structure [10,60] including various material parameters [61]. The strong electrostatic field is caused by spontaneous and piezo-electric polarization of III-nitride materials grown along the wurtzite c-axis which creates a high density of built-in net charges at all hetero-interfaces (Figure 8). Various polarization models have been published [62,63,64,65]; however, the predicted polarization charge is typically scaled down in GaN-LED simulations in order to achieve realistic results (cf. Figure 5) [14]. A possible reason for this discrepancy is the partial screening of interface polarization charges by other defects. LED growth in different, so-called non-polar or semi-polar crystal directions lowers the polarization field [11,66,67,68]. In any case, the calculated spontaneous emission spectrum often deviates from measurements which may be caused by incorrect predictions of band gap, polarization field, and/or QW structure [10]. 

Spontaneous recombination saturation effects contribute to the efficiency droop [70] as they change the balance of recombination processes in Equation (2). Microscopic models reveal that the spontaneous emission rate is proportional to n^2^ only at low current [71]. At higher current, it may be described by B = B_0_/(1 + n/n_0_) [72]. More recently, photon emission enhancement by the optical LED design has been investigated (Purcell effect) [73]. QW coupling with surface plasmons is also predicted to improve photon emission [74,75]. Nanowire LEDs allow for strain relaxation and enhanced radiative recombination [76]. Bipolar cascade LED designs are envisioned to enable RE > IQE > EQE > 1 at elevated output power as multiple active regions separated by tunnel junctions permit electrons to generate more than one photon [77]. Optical polarization effects gain relevance in AlGaN-based LEDs [78,79].

(C) Auger Recombination

**Figure 9:**
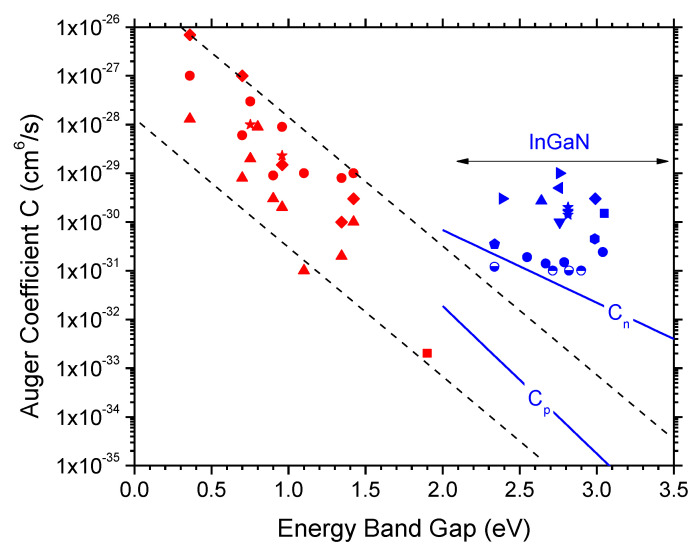
Published Auger coefficients for various semiconductors as function of energy band gap. The InGaN data (blue symbols) contradict the steep decline with larger band gap observed with other semiconductors (red symbols). The blue lines are calculated for indirect Auger excitations within conduction bands (C_n_) or valence bands (C_p_) of bulk InGaN [83].

Auger recombination is typically identified as dominating droop mechanism using ABC fits to measured efficiency vs. current characteristics [5]. However, since the Cn^3^ term in the ABC Equation (2) is the only term rising faster with carrier density than the light emission (Bn^2^), any ABC fit will hold Auger recombination responsible for the droop, no matter what the real cause is. Different models lead to different C-parameter extractions from the same measurement [42]. Figure 9 shows Auger coefficients obtained for various semiconductor materials as a function of the energy band gap. It reveals an uncertainty of several orders of magnitude accompanied by a steep decline with increasing band gap (red symbols). However, the nitride data (blue symbols) are clearly outside the broad band predicted, which caused early skepticism towards the Auger model for the efficiency droop.

Subsequently, several groups have been working on fundamental calculations of the Auger coefficient for III-nitrides. The direct Auger process—involving only three carriers—was initially determined to be very weak [80,81]. Therefore, indirect Auger recombination was proposed as a possible explanation, which includes electron–phonon coupling and alloy scattering [82,83]. However, the calculated indirect Auger coefficients are only valid for bulk layers and they are below the values required to fully explain the efficiency droop. On the other hand, the inclusion of hot Auger electron leakage in the LED model enables lower Auger parameters to cause relevant efficiency droop [84,85]. Surprisingly, other studies suggest that direct QW Auger recombination may still be strong enough, depending on QW width and composition [86]. More recent Auger recombination models include InGaN alloy disorder [87] or QW carrier localization [88,89]. Energy band structure theories are the basis of all these models, which may include too many approximations and uncertainties to deliver reliable Auger coefficients.

Direct experimental evidence for QW Auger recombination was provided by two somewhat contradicting methods. The first method measured high-energy (hot) electrons emitted from the surface layer of an LED [90]. The authors attribute these hot electrons to the QW Auger process, which facilitates electron–hole recombination by transferring the excess energy to a second electron, which thereby becomes “hot” and can travel to the LED surface. Based on Monte-Carlo simulations of this first experiment, other researchers doubt that the Auger-electron can maintain its high energy over such a long travel distance [16]. In fact, the second method assumed a very short travel distance of hot Auger electrons so that they lose their energy quickly and are captured by a neighboring quantum well [91]. However, numerical simulations of this second experiment show similar results without Auger recombination [92]. In any case, there is still much uncertainty about the physics of Auger recombination in InGaN QWs and no evidence that this is the only mechanism causing GaN-LED efficiency droop.

## 4. Light Extraction Models

The light extraction efficiency LEE in Equation (1) often imposes severe limitations on the total energy efficiency WPE. However, this problem captured relatively little attention of the GaN-LED modeling community, because it hardly contributes to the efficiency droop with higher current. Photons generated spontaneously in the active layers travel in all directions inside the LED chip. Only a fraction is able to escape from the chip, due to internal reflection and absorption. Ray tracing models are often employed to calculate LEE [93,94,95]. However, ray optics fails when structures as small as the photon wavelength are involved. In such cases, Maxwell’s equations are usually solved employing the Finite-Difference Time-Domain (FDTD) method, in particular for nano-wire LEDs [96] and photonic-crystal LEDs [97]. Tailored models have been developed for textured LED surfaces [98,99] and for the influence of Phosphor layers outside the semiconductor chip [95,100]. Some models also include photon recycling, i.e., their re-absorption by the quantum wells [101]. Light polarization effects need to be considered in deep ultraviolet AlGaN-based LEDs [102].

**Figure 10:**
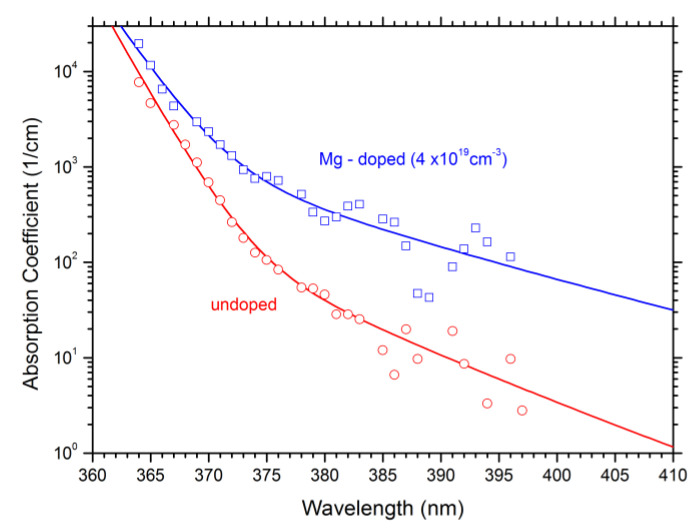
GaN absorption coefficients vs. photon wavelength with and without Mg doping. The GaN band gap wavelength is 363nm. Dots—measurement; lines—fitted band-tail model [108].

Refractive index and absorption coefficient are the two key material parameters of LEE models [93], which may also be given as real and imaginary part of the complex dielectric constant [103]. Both depend on material composition and photon wavelength. Based on available measurements, simple refractive index formulas for III-nitride alloys have been developed by several groups [10,93,104,105]. Photon absorption is more difficult to predict as it strongly depends on growth quality and doping [106,107]. In particular, the high Mg doping density is known to cause significant photon absorption, which may be attributed to disorder-induced band tails (Figure 10) [108]. 

## 5. Self-Heating Models

**Figure 11:**
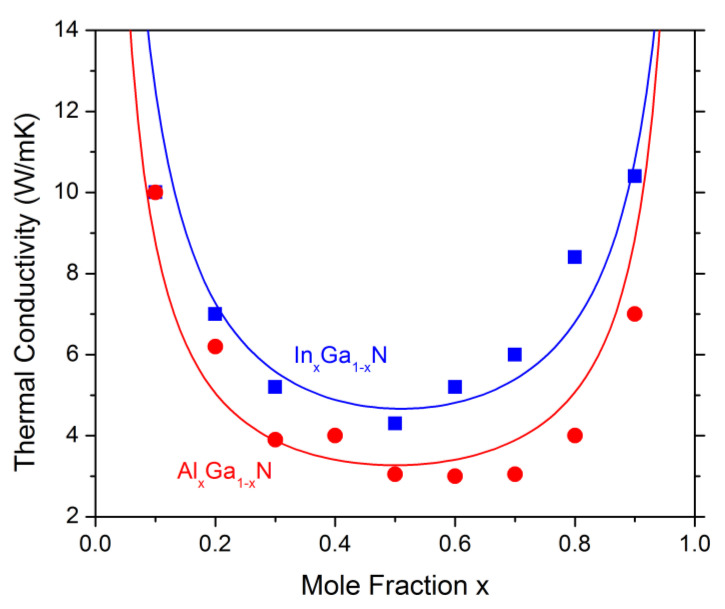
Bulk thermal conductivity as function of alloy parameter x as predicted by a molecular dynamics model [119] (dots) and fitted by an analytical function [117]. Measured values for GaN are near 100 W/mK.

The LED efficiency is known to decline with increasing chip temperature [109,110,111]. However, self-heating is a three-dimensional problem and only considered by a few self-consistent GaN-LED simulations [10,112,113]. The thermal conductivity is the key material parameter of such heat flux computations. It is relatively high in perfect GaN crystals, but drops significantly due to phonon scattering at dopants [114], defects [115], and interfaces [116]. Bulk ternary layers suffer from strong alloy scattering of phonons (Figure 11) [117]. LED chip mounting and packaging also influence the self-heating significantly [118]. Thus, there is much uncertainty about the thermal material properties of real devices so that thermal resistance measurements are often preferred over self-heating simulations.

## 6. Key Modeling and Simulation Challenges 

**Figure 12:**
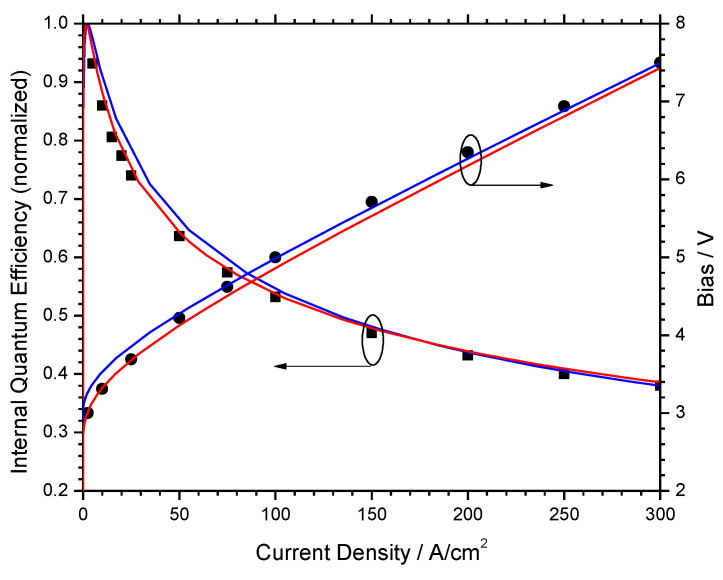
Normalized IQE and bias vs. current density (symbols—measurement; red lines—simulation favoring Auger recombination; blue lines—simulation favoring electron leakage) [29].

The strong influence of material properties discussed above indicates that the employment of realistic material parameters remains a great challenge for GaN-LED efficiency models. In fact, advanced drift-diffusion simulations of experimental characteristics were shown to validate competing efficiency droop models by simple variation of uncertain parameters [29]. Figure 12 shows good agreement with both efficiency and bias measurements (dots) by enabling dominating carrier loss from Auger recombination (red lines) or from electron leakage (blue lines). The switch was accomplished by changing the Auger coefficient C of the quantum wells and the acceptor doping density N_A_ of the electron blocking layer, both of which are unknown for real devices. High values produce dominating Auger recombination, while low values favor electron leakage in the simulation.

GaN-LEDs are three-dimensional (3D) objects, but most LED simulations are performed in 1D or 2D (cf. Figure 4). Even with uniform material properties in each semiconductor layer, the current flow is often non-uniform in real devices [10,11,15], leading to local self-heating, non-uniform carrier density in each QW, non-uniform light emission, and enhanced efficiency droop [15,120]. While 1D and 2D simulations are very valuable in studying specific mechanisms, they are unable to fully reflect the internal physics and the measured performance of real LEDs. 

Another major challenge arises from the non-uniform nature of InGaN quantum wells and other thin alloy layers [121]. QWs with low Indium content may exhibit an average Indium atom distance that is larger than the QW thickness. QWs with larger Indium concentration show Indium accumulation regions with lower bandgap, larger free carrier concentration, and stronger Auger recombination. Thus, the typical assumption of uniform QW properties is often invalid. That is why non-uniformity models have been developed in recent years, often embedded in multi-scale LED simulations [121,122,123,124,125]. However, the more inclusive an LED model is, the more uncertain parameters are usually involved which undermines the reliability of quantitative results.

Artificial intelligence methods also represent a serious challenge. Simulation-based machine learning approaches have been applied to GaN-LED design optimization [126,127] but produced unreliable results [128]. The great popularity of such methods in materials science [129] and in photonics [130] seems hard to transfer to optoelectronic devices considering their complex internal physics and their material parameter uncertainties. In fact, the strength of machine learning lies in the analysis of large amounts of experimental data which are often routinely collected in the industrial LED production. The combination of reality-trained artificial neural networks (ANNs) with numerical simulations could lead to the creation of realistic digital twins that support the LED design and production process [118,131,132].

## 7. Conclusions

Various models have been developed for almost all aspects of GaN-LED device physics that provide valuable insight into internal mechanisms affecting the energy efficiency. However, all models simplify reality so that their relevance and accuracy should always be validated by experiments. Special attention should be paid to the employment of realistic material parameters. Reliable results can only be achieved by interactive and synergetic combination of theoretical modeling, numerical simulation, and experimental investigation. Other challenges lie in the self-consistent inclusion of three-dimensional effects and of atomic-scale non-uniformities.

## References

[1] Nakamura S. (2015). Background Story of the Invention of Efficient InGaN Blue-Light-Emitting Diodes (Nobel Lecture). Angew. Chem. Int. Ed..

[2] Weisbuch C. (2020). Review—On The Search for Efficient Solid State Light Emitters: Past, Present, Future. ECS J. Solid State Sci. Technol..

[3] Taki T., Strassburg M. (2019). Review—Visible LEDs: More than Efficient Light. ECS J. Solid State Sci. Technol..

[4] Piprek J. (2010). Efficiency droop in nitride-based light-emitting diodes. Phys. Status Solidi (a).

[5] Shen Y.C., Mueller G.O., Watanabe S., Gardner N.F., Munkholm A., Krames M.R. (2007). Auger recombination in InGaN measured by photoluminescence. Appl. Phys. Lett..

[6] Kim M.-H., Schubert M.F., Dai Q., Kim J.K., Schubert E.F., Piprek J., Park Y. (2007). Origin of efficiency droop in GaN-based light-emitting diodes. Appl. Phys. Lett..

[7] Verzellesi G., Saguatti D., Meneghini M., Bertazzi F., Goano M., Meneghesso G., Zanoni E. (2013). Efficiency droop in InGaN/GaN blue light-emitting diodes: Physical mechanisms and remedies. J. Appl. Phys..

[8] Usman M., Anwar A.-R., Munsif M. (2020). Review—A Survey of Simulations on Device Engineering of GaN-Based Light-Emitting Diodes. ECS J. Solid State Sci. Technol..

[9] Shim J.-I., Shin D.-S., Oh C.-H., Jung H. (2019). Review—Active Efficiency as a Key Parameter for Understanding the Efficiency Droop in InGaN-Based Light-Emitting Diodes. ECS J. Solid State Sci. Technol..

[10] Piprek J., Li S., Piprek J. (2005). GaN-based Light-Emitting Diodes. Optoelectronic Devices: Advanced Simulation and Analysis.

[11] Karpov S.Y., Piprek J. (2017). Light-Emitting Diode Fundamentals. Handbook of Optoelectronic Device Modeling and Simulation.

[12] Römer F., Witzigmann B. (2015). Acceptor activation model for III-nitride LEDs. J. Comput. Electron..

[13] Bellotti E., Bertazzi F., Piprek J. (2007). Transport Parameters for Electrons and Holes. Nitride Semiconductor Devices: Principles and Simulation.

[14] Piprek J. (2016). Comparative efficiency analysis of GaN-based light-emitting diodes and laser diodes. Appl. Phys. Lett..

[15] Zhou S., Liu X., Yan H., Chen Z., Liu Y., Liu S. (2019). Highly efficient GaN-based high-power flip-chip light-emitting diodes. Opt. Express.

[16] Bertazzi F., Goano M., Zhou X., Calciati M., Ghione G., Matsubara M., Bellotti E. (2015). Looking for Auger signatures in III-nitride light emitters: A full-band Monte Carlo perspective. Appl. Phys. Lett..

[17] Kivisaari P., Sadi T., Li J., Rinke P., Oksanen J. (2017). On the Monte Carlo Description of Hot Carrier Effects and Device Characteristics of III-N LEDs. Adv. Electron. Mater..

[18] Der Maur M.A. (2015). Multiscale approaches for the simulation of InGaN/GaN LEDs. J. Comput. Electron..

[19] Geng J., Sarangapani P., Wang K.-C., Nelson E., Browne B., Wordelman C., Charles J., Chu Y., Kubis T., Klimeck G. (2017). Quantitative Multi-Scale, Multi-Physics Quantum Transport Modeling of GaN-Based Light Emitting Diodes. Phys. Status Solidi (a).

[20] Wang K.-C., Grassi R., Chu Y., Sureshbabu S.H., Geng J., Sarangapani P., Guo X., Townsend M., Kubis T. (2020). Introduction of multi-particle Büttiker probes—Bridging the gap between drift diffusion and quantum transport. J. Appl. Phys..

[21] Piprek J., Li Z.M.S. (2013). Origin of InGaN light-emitting diode efficiency improvements using chirped AlGaN multi-quantum barriers. Appl. Phys. Lett..

[22] Mandurrino M., Verzellesi G., Goano M., Vallone M., Bertazzi F., Ghione G., Meneghini M., Meneghesso G., Zanoni E. (2015). Physics-based modeling and experimental implications of trap-assisted tunneling in InGaN/GaN light-emitting diodes. Phys. Status Solidi (a).

[23] Piprek J. (2014). Blue light emitting diode exceeding 100% quantum efficiency. Phys. Status Solidi (RRL) Rapid Res. Lett..

[24] Kuo Y.-K., Chang J.-Y., Shih Y.-H., Chen F.-M., Tsai M.-C., Piprek J., Piprek J. (2017). Tunnel-Junction Light-Emitting Diodes. Handbook of Optoelectronic Device Modeling and Simulation.

[25] Lin Y.-Y., Chuang R.W., Chang S., Li S., Jiao Z.-Y., Ko T.-K., Hon S.J., Liu C.H. (2012). GaN-Based LEDs With a Chirped Multiquantum Barrier Structure. IEEE Photon.-Technol. Lett..

[26] Kivisaari P., Oksanen J., Tulkki J., Sadi T. (2015). Monte Carlo simulation of hot carrier transport in III-N LEDs. J. Comput. Electron..

[27] Li S. (2015). Non-local transport in numerical simulation of GaN LED. J. Comput. Electron..

[28] Wu C.-K., Li C.-K., Wu Y.-R. (2015). Percolation transport study in nitride based LED by considering the random alloy fluctuation. J. Comput. Electron..

[29] Piprek J. (2015). How to decide between competing efficiency droop models for GaN-based light-emitting diodes. Appl. Phys. Lett..

[30] Guo X., Schubert E.F. (2001). Current crowding and optical saturation effects in GaInN/GaN light-emitting diodes grown on insulating substrates. Appl. Phys. Lett..

[31] Piprek J. (2003). Semiconductor Optoelectronic Devices.

[32] Hurni C.A., David A., Cich M.J., Aldaz R.I., Ellis B., Huang K., Tyagi A., Delille R.A., Craven M.D., Steranka F. (2015). Bulk GaN flip-chip violet light-emitting diodes with optimized efficiency for high-power operation. Appl. Phys. Lett..

[33] Piprek J., Li Z.-M. (2016). Electroluminescent cooling mechanism in InGaN/GaN light-emitting diodes. Opt. Quantum Electron..

[34] Sadi T., Radevici I., Oksanen J. (2020). Thermophotonic cooling with light-emitting diodes. Nat. Photon..

[35] De Santi C., Meneghini M., Tibaldi A., Vallone M., Goano M., Bertazzi F., Verzellesi G., Meneghesso G., Zanoni E. (2018). Physical mechanisms limiting the performance and the reliability of GaN-based LEDs. Nitride Semiconductor Light-Emitting Diodes (LEDs).

[36] Lin G.-B., Meyaard D., Cho J., Schubert E.F., Shim H., Sone C. (2012). Analytic model for the efficiency droop in semiconductors with asymmetric carrier-transport properties based on drift-induced reduction of injection efficiency. Appl. Phys. Lett..

[37] Piprek J., Li S. (2010). Electron leakage effects on GaN-based light-emitting diodes. Opt. Quantum Electron..

[38] Piprek J., Li S. (2013). Sensitivity analysis of electron leakage in III-nitride light-emitting diodes. Appl. Phys. Lett..

[39] Ahn B.-J., Kim T.-S., Dong Y., Hong M.-T., Song J.-H., Song J.-H., Yuh H.-K., Choi S.-C., Bae D.-K., Moon Y. (2012). Experimental determination of current spill-over and its effect on the efficiency droop in InGaN/GaN blue-light-emitting-diodes. Appl. Phys. Lett..

[40] Jung E., Hwang G., Chung J., Kwon O., Han J., Moon Y.-T., Seong T.-Y. (2015). Investigating the origin of efficiency droop by profiling the temperature across the multi-quantum well of an operating light-emitting diode. Appl. Phys. Lett..

[41] David A., Young N.G., Lund C., Craven M.D. (2020). Review—The Physics of Recombinations in III-Nitride Emitters. ECS J. Solid State Sci. Technol..

[42] Piprek J., Römer F., Witzigmann B. (2015). On the uncertainty of the Auger recombination coefficient extracted from InGaN/GaN light-emitting diode efficiency droop measurements. Appl. Phys. Lett..

[43] Ryu H.-Y., Shin D.-S., Shim J.-I. (2012). Analysis of efficiency droop in nitride light-emitting diodes by the reduced effective volume of InGaN active material. Appl. Phys. Lett..

[44] Ryu H.-Y. (2020). Modeling and analysis of the effects of inhomogeneous carrier distributions in InGaN multiple quantum wells. Curr. Appl. Phys..

[45] Zhao L., Yan D., Zhang Z., Hua B., Yang G., Cao Y., Zhang E.X., Gu X., Fleetwood D.M. (2018). Temperature-Dependent Efficiency Droop in GaN-Based Blue LEDs. IEEE Electron Device Lett..

[46] Sarkissian R., Roberts S.T., Yeh T.-W., Das S., Bradforth S., O’Brien J., Dapkus P.D. (2013). Photon quenching in InGaN quantum well light emitting devices. Appl. Phys. Lett..

[47] Modine N.A., Armstrong A.M., Crawford M.H., Chow W.W. (2013). Highly nonlinear defect-induced carrier recombination rates in semiconductors. J. Appl. Phys..

[48] Espenlaub A.C., Myers D.J., Young E., Marcinkevičius S., Weisbuch C., Speck J.S. (2019). Evidence of trap-assisted Auger recombination in low radiative efficiency MBE-grown III-nitride LEDs. J. Appl. Phys..

[49] Usman M., Anwar A.-R., Munsif M., Malik S., Islam N.U. (2019). Analytical analysis of internal quantum efficiency with polarization fields in GaN-based light-emitting diodes. Superlattices Microstruct..

[50] Han D.-P., Lee G.W., Min S., Shin D.-S., Shim J.-I., Iwaya M., Takeuchi T., Kamiyama S., Akasaki I. (2020). Identifying the cause of thermal droop in GaInN-based LEDs by carrier-and thermo-dynamics analysis. Sci. Rep..

[51] Ryu H.-Y., Ryu G.-H., Onwukaeme C., Ma B. (2020). Temperature dependence of the Auger recombination coefficient in InGaN/GaN multiple-quantum-well light-emitting diodes. Opt. Express.

[52] Karpov S. (2014). ABC-model for interpretation of internal quantum efficiency and its droop in III-nitride LEDs: A review. Opt. Quantum Electron..

[53] Karpov S.Y., Piprek J. (2007). Visible Light-Emitting Diodes. Nitride Semiconductor Devices: Principles and Simulation.

[54] Hangleiter A., Netzel C., Fuhrmann D., Hitzel F., Hoffmann L., Bremers H., Rossow U., Adé G., Hinze P. (2007). Anti-localization suppresses non-radiative recombination in GaInN/GaN quantum wells. Philos. Mag..

[55] Hader J., Moloney J.V., Koch S.W. (2010). Density-activated defect recombination as a possible explanation for the efficiency droop in GaN-based diodes. Appl. Phys. Lett..

[56] Karpov S. (2010). Effect of localized states on internal quantum efficiency of III-nitride LEDs. Phys. Status Solidi (RRL) Rapid Res. Lett..

[57] Chow W.W. (2011). Modeling excitation-dependent bandstructure effects on InGaN light-emitting diode efficiency. Opt. Express.

[58] David A., Hurni C.A., Young N.G., Craven M.P. (2017). Field-assisted Shockley-Read-Hall recombinations in III-nitride quantum wells. Appl. Phys. Lett..

[59] Kisin M.V., El-Ghoroury H.S. (2013). Modeling of III-Nitride Multiple-Quantum-Well Light-Emitting Structures. IEEE J. Sel. Top. Quantum Electron..

[60] Bulashevich K., Mymrin V., Karpov S., Zhmakin I., Zhmakin A. (2006). Simulation of visible and ultra-violet group-III nitride light emitting diodes. J. Comput. Phys..

[61] Vurgaftman I., Meyer J.R. (2003). Band parameters for nitrogen-containing semiconductors. J. Appl. Phys..

[62] Fiorentini V., Bernardini F., Ambacher O. (2002). Evidence for nonlinear macroscopic polarization in III–V nitride alloy heterostructures. Appl. Phys. Lett..

[63] Bernardini F.T., Piprek J. (2007). Spontaneous and Piezoelectric Polarization: Basic Theory vs. Practical Recipes. Nitride Semiconductor Devices: Principles and Simulation.

[64] Caro M.A., Schulz S., O’Reilly E.P. (2013). Theory of local electric polarization and its relation to internal strain: Impact on polarization potential and electronic properties of group-III nitrides. Phys. Rev. B.

[65] Migliorato M.A., Pal J., Huang X., Hu W., Willatzen M., Gu Y., Piprek J., Piprek J. (2017). Polarization in III-N Semiconductors. Handbook of Optoelectronic Device Modeling and Simulation.

[66] Romanov A.E., Baker T.J., Nakamura S., Speck J.S. (2006). ERATO/JST UCSB Group Strain-induced polarization in wurtzite III-nitride semipolar layers. J. Appl. Phys..

[67] Schulz S., Marquardt O. (2015). Electronic Structure of Polar and Semipolar (112¯2)-Oriented Nitride Dot-in-a-Well Systems. Phys. Rev. Appl..

[68] Monavarian M., Rashidi A., Feezell D. (2018). A Decade of Nonpolar and Semipolar III-Nitrides: A Review of Successes and Challenges. Phys. Status Solidi (a).

[69] Piprek J. (2011). Ultra-violet light-emitting diodes with quasi acceptor-free AlGaN polarization doping. Opt. Quantum Electron..

[70] Shim J.-I., Kim H., Shin D.-S., Yoo H.-Y. (2011). An Explanation of Efficiency Droop in InGaN-based Light Emitting Diodes: Saturated Radiative Recombination Rate at Randomly Distributed In-Rich Active Areas. J. Korean Phys. Soc..

[71] Hader J., Moloney J.V., Koch S. (2005). Supression of carrier recombination in semiconductor lasers by phase-space filling. Appl. Phys. Lett..

[72] David A., Grundmann M.J. (2010). Droop in InGaN light-emitting diodes: A differential carrier lifetime analysis. Appl. Phys. Lett..

[73] Choi Y.H., Ryu G.H., Ryu H.Y. (2017). Numerical Investigation of Purcell Enhancement of the Internal Quantum Efficiency of GaN-based Green LED Structures. Curr. Opt. Photon..

[74] Kuo Y., Chang W.-Y., Chen H.-S., Wu Y.-R., Yang C.-C.C.C., Kiang Y.-W. (2013). Surface-plasmon-coupled emission enhancement of a quantum well with a metal nanoparticle embedded in a light-emitting diode. J. Opt. Soc. Am. B.

[75] Chang W.-Y., Kuo Y., Kiang Y.-W., Yang C.-C. (2019). Simulation study on light color conversion enhancement through surface plasmon coupling. Opt. Express.

[76] Sacconi F., Piprek J. (2017). Quantum Disk Nanowire Light-Emitting Diodes. Handbook of Optoelectronic Device Modeling and Simulation.

[77] Piprek J. (2014). GaN-based bipolar cascade light-emitting diode with 250% peak quantum efficiency. Opt. Quantum Electron..

[78] Durnev M.V., Karpov S.Y. (2013). Polarization phenomena in light emission from C -plane Al(In)GaN heterostructures. Phys. Status Solidi (b).

[79] Lu H., Yu T., Yuan G., Jia C., Chen G., Zhang G. (2012). Valence subband coupling effect on polarization of spontaneous emissions from Al-rich AlGaN/AlN quantum wells. Opt. Express.

[80] Hader J., Moloney J.V., Pasenow B., Koch S., Sabathil M., Linder N., Lutgen S. (2008). On the importance of radiative and Auger losses in GaN-based quantum wells. Appl. Phys. Lett..

[81] Bertazzi F., Goano M., Bellotti E. (2010). A numerical study of Auger recombination in bulk InGaN. Appl. Phys. Lett..

[82] Kioupakis E., Rinke P., Delaney K.T., Van De Walle C.G. (2011). Indirect Auger recombination as a cause of efficiency droop in nitride light-emitting diodes. Appl. Phys. Lett..

[83] Bertazzi F., Goano M., Bellotti E. (2012). Numerical analysis of indirect Auger transitions in InGaN. Appl. Phys. Lett..

[84] Deppner M., Römer F., Witzigmann B. (2012). Auger carrier leakage in III-nitride quantum-well light emitting diodes. Phys. Status Solidi (RRL) Rapid Res. Lett..

[85] Sadi T., Kivisaari P., Oksanen J., Tulkki J. (2014). On the correlation of the Auger generated hot electron emission and efficiency droop in III-N light-emitting diodes. Appl. Phys. Lett..

[86] Bertazzi F., Zhou X., Goano M., Ghione G., Bellotti E. (2013). Auger recombination in InGaN/GaN quantum wells: A full-Brillouin-zone study. Appl. Phys. Lett..

[87] Zinovchuk A., Gryschuk A.M. (2018). Alloy-assisted Auger recombination in InGaN. Opt. Quantum Electron..

[88] Shahmohammadi M., Liu W., Rossbach G., Lahourcade L., Dussaigne A., Bougerol C., Butté R., Grandjean N., Deveaud B., Jacopin G. (2017). Enhancement of Auger recombination induced by carrier localization in InGaN/GaN quantum wells. Phys. Rev. B.

[89] Jones C.M., Teng C.-H., Yan Q., Ku P.-C., Kioupakis E. (2017). Impact of carrier localization on recombination in InGaN quantum wells and the efficiency of nitride light-emitting diodes: Insights from theory and numerical simulations. Appl. Phys. Lett..

[90] Iveland J., Martinelli L., Peretti J., Speck J.S., Weisbuch C. (2013). Direct Measurement of Auger Electrons Emitted from a Semiconductor Light-Emitting Diode under Electrical Injection: Identification of the Dominant Mechanism for Efficiency Droop. Phys. Rev. Lett..

[91] Binder M., Nirschl A., Zeisel R., Hager T., Lugauer H.-J., Sabathil M., Bougeard D., Wagner J., Galler B. (2013). Identification of nnp and npp Auger recombination as significant contributor to the efficiency droop in (GaIn)N quantum wells by visualization of hot carriers in photoluminescence. Appl. Phys. Lett..

[92] Hader J., Moloney J.V., Koch S.W. (2015). Optical excitation dependent emission properties of InGaN quantum wells. J. Comput. Electron..

[93] Zabelin V., Zakheim D., Gurevich S. (2004). Efficiency improvement of AlGaInN LEDs advanced by ray-tracing analysis. IEEE J. Quantum Electron..

[94] Liu Z., Wang K., Luo X., Liu S. (2010). Precise optical modeling of blue light-emitting diodes by Monte Carlo ray-tracing. Opt. Express.

[95] Kashiwao T., Hiura M., Ikeda K., Deguchi M., Bahadori A. (2018). Investigation of effects of phosphor particles on optimal design of surface-mount-device light-emitting diode packaging using ray-tracing simulation. IET Optoelectron..

[96] Lin R., Galan S.V., Sun H., Hu Y., Alias M.S., Janjua B., Ng T., Ooi B., Li X. (2018). Tapering-induced enhancement of light extraction efficiency of nanowire deep ultraviolet LED by theoretical simulations. Photon. Res..

[97] Wiesmann C., Bergenek K., Linder N., Schwarz U.T. (2009). Photonic crystal LEDs - designing light extraction. Laser Photon. Rev..

[98] Pan J.-W., Tsai P.-J., Chang K.-D., Chang Y.-Y. (2013). Light extraction efficiency analysis of GaN-based light-emitting diodes with nanopatterned sapphire substrates. Appl. Opt..

[99] David A. (2013). Surface-Roughened Light-Emitting Diodes: An Accurate Model. J. Disp. Technol..

[100] Linder N., Eisert D., Jermann F., Piprek J. (2007). Simulation of LEDs with Phosphorescent Media for the Generation of White Light. Nitride Semiconductor Devices: Principles and Simulation.

[101] David A., Hurni C.A., Aldaz R.I., Cich M.J., Ellis B., Huang K., Steranka F., Krames M.R. (2014). High light extraction efficiency in bulk-GaN based volumetric violet light-emitting diodes. Appl. Phys. Lett..

[102] Kim D.Y., Park J.H., Lee J.W., Hwang S., Oh S.J., Kim J., Sone C., Schubert E.F., Kim J.K. (2015). Overcoming the fundamental light-extraction efficiency limitations of deep ultraviolet light-emitting diodes by utilizing transverse-magnetic-dominant emission. Light. Sci. Appl..

[103] Goldhahn R., Buchheim C., Schley P., Winzer A.T., Wenzel H., Piprek J. (2007). Optical Constants of Bulk Nitrides. Nitride Semiconductor Devices: Principles and Simulation.

[104] Laws G.M., Larkins E.C., Harrison I., Molloy C., Somerford D. (2001). Improved refractive index formulas for the AlxGa1−xN and InyGa1−yN alloys. J. Appl. Phys..

[105] Alam S.N., Zubialevich V.Z., Ghafary B., Parbrook P.J. (2020). Bandgap and refractive index estimates of InAlN and related nitrides across their full composition ranges. Sci. Rep..

[106] Kioupakis E., Rinke P., Van De Walle C.G. (2010). Determination of Internal Loss in Nitride Lasers from First Principles. Appl. Phys. Express.

[107] Sizov D., Bhat R., Zah C.-E. (2013). Optical absorption of Mg-doped layers and InGaN quantum wells on c-plane and semipolar GaN structures. J. Appl. Phys..

[108] Piprek J., Wenzel H., Kneissl M. (2007). Analysis of wavelength-dependent performance variations of GaN-based ultraviolet lasers. Optoelectronic Devices: Physics, Fabrication, and Application IV, Proceedings of the Optics East, Boston, MA, USA, 12 October 2007.

[109] Li Y., Zhu Y., Huang J., Deng H., Wang M., Yin H. (2017). The effects of temperature on optical properties of InGaN/GaN multiple quantum well light-emitting diodes. J. Appl. Phys..

[110] Meneghini M., De Santi C., Tibaldi A., Vallone M., Bertazzi F., Meneghesso G., Zanoni E., Goano M. (2020). Thermal droop in III-nitride based light-emitting diodes: Physical origin and perspectives. J. Appl. Phys..

[111] David A., Young N.G., Lund C., Craven M.P. (2019). Thermal droop in high-quality InGaN LEDs. Appl. Phys. Lett..

[112] Bogdanov M.V., Bulashevich K.A., Evstratov I.Y., Zhmakin A.I., Karpov S. (2008). Coupled modeling of current spreading, thermal effects and light extraction in III-nitride light-emitting diodes. Semicond. Sci. Technol..

[113] Chernyakov A.E., Bulashevich K.A., Karpov S.Y., Zakgeim A.L. (2013). Experimental and theoretical study of electrical, thermal, and optical characteristics of InGaN/GaN high-power flip-chip LEDs. Phys. Status solidi (a).

[114] Florescu D.I., Asnin V.M., Pollak F.H., Molnar R.J., Wood C.E.C. (2000). High spatial resolution thermal conductivity and Raman spectroscopy investigation of hydride vapor phase epitaxy grown n-GaN/sapphire (0001): Doping dependence. J. Appl. Phys..

[115] Zou J., Kotchetkov D., Balandin A.A., Florescu D.I., Pollak F.H. (2002). Thermal conductivity of GaN films: Effects of impurities and dislocations. J. Appl. Phys..

[116] Ziade E., Yang J., Brummer G., Nothern D., Moustakas T., Schmidt A.J. (2017). Thickness dependent thermal conductivity of gallium nitride. Appl. Phys. Lett..

[117] Piprek J., Piprek J. (2007). Introduction: Thermal Parameters. Nitride Semiconductor Devices: Principles and Simulation.

[118] Van Der Schans M., Yu J., Martin G. (2020). Digital Luminaire Design Using LED Digital Twins—Accuracy and Reduced Computation Time: A Delphi4LED Methodology. Energies.

[119] Kawamura T., Kangawa Y., Kakimoto K. (2006). Investigation of thermal conductivity of nitride mixed crystals and superlattices by molecular dynamics. Phys. Status Solidi (c).

[120] Ryu H.-Y., Shim J.-I. (2011). Effect of current spreading on the efficiency droop of InGaN light-emitting diodes. Opt. Express.

[121] Wu C.-K., Yang T.-J., Wu Y.-R., Piprek J., Piprek J. (2017). Influence of Random InGaN Alloy Fluctuations on GaN-Based Light-Emitting Diodes. Handbook of Optoelectronic Device Modeling and Simulation.

[122] Li C.-K., Piccardo M., Lu L.-S., Mayboroda S., Martinelli L., Peretti J., Speck J., Weisbuch C., Filoche M., Wu Y.-R. (2017). Localization landscape theory of disorder in semiconductors. III. Application to carrier transport and recombination in light emitting diodes. Phys. Rev. B.

[123] Chen H.-H., Speck J., Weisbuch C., Wu Y.-R. (2018). Three dimensional simulation on the transport and quantum efficiency of UVC-LEDs with random alloy fluctuations. Appl. Phys. Lett..

[124] O’Donovan M., Luisier M., O’Reilly E.P., Schulz S. (2020). Impact of Random Alloy Fluctuations on inter-well transport in InGaN/GaN multi-quantum well systems: An atomistic non-equilibrium Green’s function study. J. Phys. Condens. Matter.

[125] Di Vito A., Pecchia A., Di Carlo A., Der Maur M.A. (2020). Simulating random alloy effects in III-nitride light emitting diodes. J. Appl. Phys..

[126] Kim D.Y., Lin G.-B., Hwang S., Park J.H., Meyaard D., Schubert E.F., Ryu H.-Y., Kim J.K. (2015). Polarization-Engineered High-Efficiency GaInN Light-Emitting Diodes Optimized by Genetic Algorithm. IEEE Photon. J..

[127] Rouet-Leduc B., Barros K., Lookman T., Humphreys C.J. (2016). Optimisation of GaN LEDs and the reduction of efficiency droop using active machine learning. Sci. Rep..

[128] Piprek J. (2020). Pitfalls of simulation-based machine learning in optoelectronic device design. TechRxiv Prepr..

[129] Luo S., Li T., Wang X., Faizan M., Zhang L. (2020). High-throughput computational materials screening and discovery of optoelectronic semiconductors. Wiley Interdiscip. Rev. Comput. Mol. Sci..

[130] Ma W., Liu Z., Kudyshev Z.A., Boltasseva A., Cai W., Liu Y. (2020). Deep learning for the design of photonic structures. Nat. Photonics.

[131] Ibrahim M.S., Fan J., Yung W.K.C., Prisacaru A., van W., Fan X., Zhang G. (2020). Machine Learning and Digital Twin Driven Diagnostics and Prognostics of Light-Emitting Diodes. Laser Photonics Rev..

[132] Wagner-Mohnsen H., Altermatt P. (2020). A Combined Numerical Modeling and Machine Learning Approach for Optimization of Mass-Produced Industrial Solar Cells. IEEE J. Photovolt..

